# The -607C/A Polymorphisms in Interleukin-18 Gene Promoter Contributes to Cancer Risk: Evidence from A Meta-Analysis of 22 Case-Control Studies

**DOI:** 10.1371/journal.pone.0076915

**Published:** 2013-10-09

**Authors:** Ming Wang, Xiao-Yang Zhu, Liang Wang, Yu Lin

**Affiliations:** 1 Department of Radiation Therapy, Cangzhou Central Hospital, Cangzhou, Hebei Province, China; 2 Department of Radiation Therapy, the Second Affiliated Hospital of Zhejiang University School of Medicine, Hangzhou, Zhejiang Province, China; 3 Department of Endoscopic Professional, Cangzhou Central Hospital, Cangzhou, Hebei Province, China; 4 Department of Radiation Therapy, the Affiliated Hospital of Inner Mongolia Medical University, Hohhot, Neimenggu Province, China; Duke Cancer Institute, United States of America

## Abstract

**Background:**

Several observational studies have investigated the association between -607 C/A polymorphism of IL-18 gene and cancer risk; however, the results were inconsistent. Therefore, we performed a meta-analysis to derive a more precise estimation of the association to help us better understand the relationship between -607 C/A polymorphism of IL-18 gene promoter and risk of cancer.

**Methods:**

A literature search was carried out using PubMed, EMBASE, and China National Knowledge Infrastructure (CNKI) database between January 1966 and February 2013. Fixed-effect and random-effect models were used to estimate the pooled odds ratio (OR) and the corresponding 95% confidence intervals (CIs).

**Results:**

A total of 22 case-control studies including 4100 cancer cases and 4327 controls contributed to the analysis. Significant association between -607C/A polymorphism in IL-18 gene promoter and cancer risk was observed (CA vs CC:OR =1.221, 95% CI: 1.096, 1.360; P_heterogeneity_=0.219; AA/CA vs. CC:OR =1.203, 95% CI: 1.057, 1.369; P_heterogeneity_=0.064). In the subgroup analysis by ethnicity, -607C/A polymorphism significantly increased risk of cancer among Asian population (AA/CA vs. CC:OR =1.197, 95% CI: 1.023,1.401; P_heterogeneity_=0.088); however, no significant association was found in Caucasian or African population. The -607C/A polymorphism was associated with a significantly increased risk of nasopharyngeal carcinoma (CA vs CC:OR =1.330, 95% CI: 1.029,1.719; P_heterogeneity_=0.704; AA/CA vs. CC:OR =1.323, 95% CI: 1.037,1.687; P_heterogeneity_=0.823) and esophageal cancer (AA/CA vs. CC:OR =1.289, 95% CI: 1.002,1.658; P_heterogeneity_=0.700).

**Conclusions:**

The present meta-analysis suggests that the -607C/A polymorphisms in IL-18 gene promoter is associated with a significantly increased risk of cancer, especially for nasopharyngeal carcinoma and esophageal cancer and in Asian population. More studies with larger sample size, well controlled confounding factors are warranted to validate this association.

## Introduction

IL-1 family includes ten known members, all of which are characterized by gene structure, predicted three-dimensional fold, processing, receptor, signal transduction pathway and pro-inflammatory properties [1]. IL-18, also known as interferon-gamma inducing factor (IGIF), is a member of the IL-1 super-family [2]. IL-18 is secreted by a wide range of cells, including T and B lymphocytes, and antigen-presenting cells (APCs), including activated monocytes, macrophages, Kupffer cells, Langerhans cells, and NK cells [3-5]. IL-1 beta converting enzyme can convert IL-18 to a mature biologically active 18.3-kDa form through cleavage of the propeptide. IL-18 binds to the cell through a specific receptor, IL-18R, belonging to the toll-like receptor family [6]. IL-18 plays a central role in inflammation and immune response, and is generally acknowledged as a key defense cytokine against infectious agents. Because immune stimulating effects of IL-18 have also antineoplastic properties, it was tempting to propose IL-18 as a novel adjuvant therapy against cancer [7]. A number of single nucleotide polymorphisms (SNPs) of IL-18 gene have been identified and investigated [8]. There are three SNPs in the promoter region of IL-18 gene: -137, -607 and -656, relative to the transcriptional start site, which may alter the expression of IL-18 [9]. The C to A substitution at position −607 disrupts a consensus cAMP-responsive element protein-binding site, causing altered transcription factor binding and gene expression [9]. Several observational studies have investigated the association between -607 C/A polymorphism of IL-18 gene promoter and cancer risk; however, the results were inconsistent. For example, some studies found that -607 C/A polymorphism of IL-18 gene promoter was associated with increased risk of nasopharyngeal carcinoma [10] and lung cancer [11]. However, other studies found there was no association between -607 C/A polymorphism of IL-18 gene and risk of breast cancer [12] or head and neck squamous cell carcinoma [13]. Therefore, we performed a meta-analysis to derive a more precise estimation of the association to help us better understand the relationship between -607 C/A polymorphism of IL-18 gene and risk of cancer.

## Methods

### Identification of studies

Comprehensive searches were carried out using PubMed, EMBASE, and China National Knowledge Infrastructure (CNKI) databases between January 1966 and February 2013. There were no restriction of origin and languages. Search terms included: "Interleukin-18" or "IL-18" or "rs1946518" in combination with “polymorphism” or “variant” and ‘‘cancer’’ or ‘‘neoplasm’’ or ‘‘malignancy’’. The reference list of each comparative study and previous reviews were manually examined to ﬁnd additional relevant studies.

### Inclusion and exclusion criteria

Studies were selected according to the following inclusion criteria: (i) case-control studies; (ii) investigating the association between IL-18 rs1946518 (C>A) SNP and cancer risk; (iii)cancers diagnosed by histopathology; (iiii) providing detail genotype frequencies. Studies without detail genotype frequencies were excluded. Titles and abstracts of searching results were screened and full text papers were further evaluated to confirm eligibility. Two reviewers (WM and ZXY) independently selected eligible trials. Disagreement between the two reviewers was settled by discussing with the third reviewer(WL).

### Data extraction

In the present study, the following characteristics were collected by two reviewers (WM and LY) independently using a purpose-designed form: name of first author, publishing time, country where the study was conducted, ethnicity, cancer types, source of control, number of cases and controls, genotype frequency in cases and controls. Different ethnicity descents were categorized as Asian, Caucasian, and African. Cancer types were classified as prostate cancer, esophageal cancer, nasopharyngeal carcinoma, colorectal cancer, breast cancer, cervical cancer, and other cancers (bladder cancer, renal cell carcinoma, head and neck squamous cell carcinoma, lung cancer, stomach cancer, ovarian cancer, choriocarcinoma, and oral cancer). Eligible studies were defined as hospital-based (HB) and population-based (PB) according to the control source.

### Statistical analysis

Chi-square based Q test was used to check the statistical heterogeneity between studies, and the heterogeneity was considered significant when p<0.10 [14]. The fixed-effects model (based on Mantel-Haenszel method) and random-effects model (based on DerSimonian-Laird method) were used to pool the data from different studies. The fixed-effects model was used when there was no significant heterogeneity; otherwise, the random-effects model was applied [15]. The association strength between -607 C/A (rs1946518) polymorphism and cancer risk was measured by odds ratio (OR) with 95% confidence intervals (95% CI). The estimates of pooled ORs were achieved by calculating a weighted average of OR from each study. A 95% CI was used for statistical significance test and a 95% CI without 1 for OR indicating a significant increased or reduced cancer risk. The pooled ORs were calculated for homozygote comparison (AA versus CC), heterozygote comparison (CA versus CC), dominant (CA/AA versus CC) and recessive (AA versus CC/CA) modes, assuming dominant and recessive effects of the variant A allele, respectively. Subgroup analyses were performed according to (i) cancer types, (ii) ethnicities, (iii) source of control, and (iiii) sample size, to examine the impact of these factors on the association. To test the robustness of association, sensitivity analysis were carried out by excluding studies one-by-one and analyzing the effect size for all of rest studies. Cumulative meta-analysis was also performed to identify the change in trend of reporting risk over time. In cumulative meta-analysis, studies were chronologically ordered by publication year, then the pooled RRs were obtained at the end of each year. To better investigate the possible sources of between-study heterogeneity, a meta-regression analysis was performed [16]. Publication bias was assessed using Begg and Mazumdar adjusted rank correlation test and the Egger regression asymmetry test [17,18]. HWE(Hardy-Weinberg equilibrium) was tested by Pearson’s X^2^ test (P<0.05 means deviated from HWE). All analyses were performed using Stata version 11.0 (StataCorp, College Station, TX).

## Results

### Search results and characteristics of studies included in the meta-analysis

**Figure 1:**
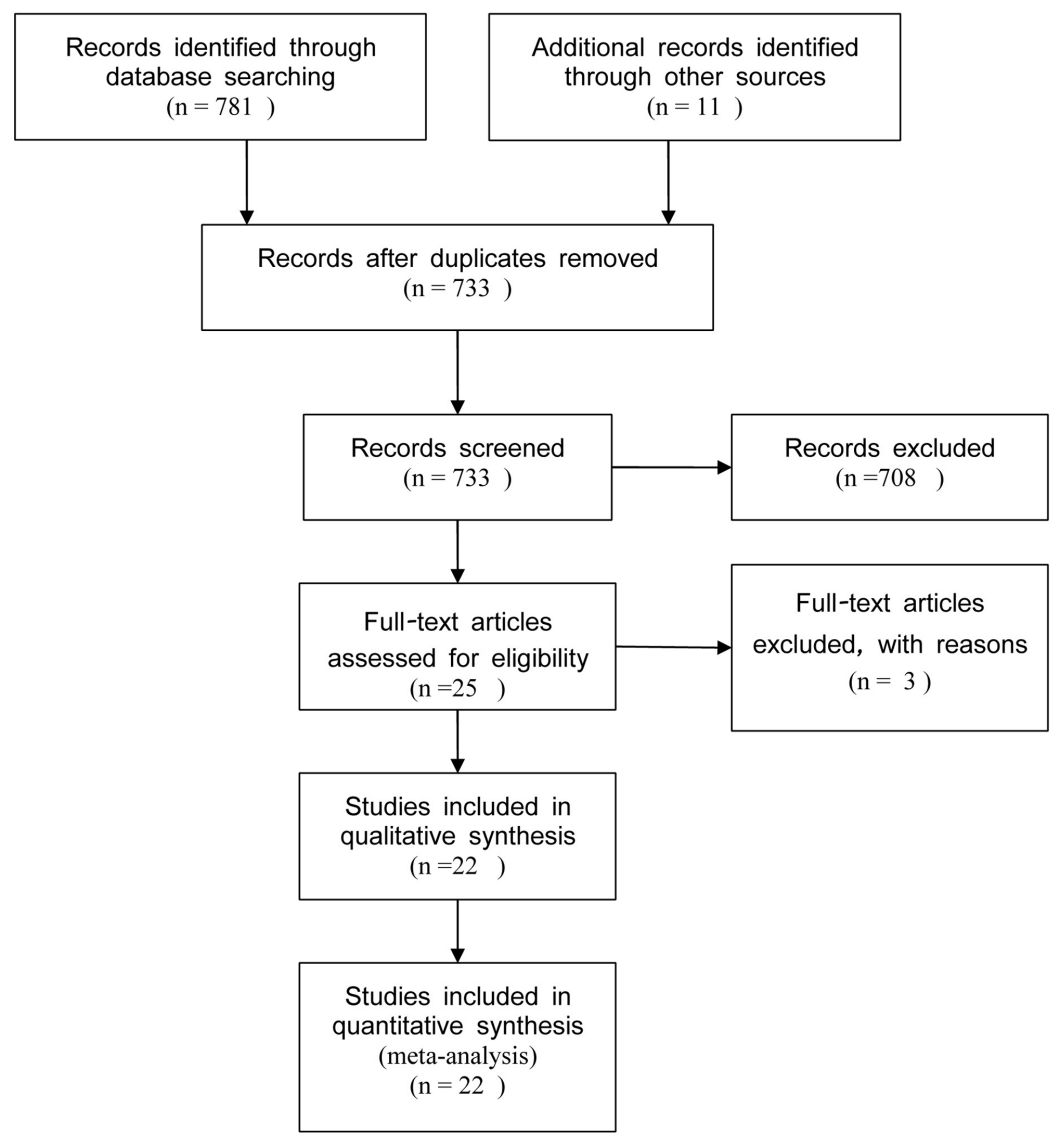
Flow diagram of the study selection process.

A total of 792 citations were identified during the initial search (shown in Figure 1). On the basis of the title and abstract, we identified 24 papers. After detailed evaluation, one study was excluded for incorrect data, and two studies were excluded for having not presented -607 C/A polymorphisms. In the study reported by Haghshenas MR and colleagues [19], they investigated rs1946518 polymorphisms and colorectal cancer, as well as stomach cancer, and the data was presented separately, thus both of them were considered as a separate study in this meta-analysis. At last, 22 case-control studies [10-13,19-35], including 4100 cancer cases and 4327 controls, were included in the meta-analysis(Baseline data and other details are shown in Table 1). 16 eligible studies were conducted in Asia [11-13,19,21,23-27,29,30,32,34,35], five in Europe [10,20,28,31,33], and the remaining one in Africa [22]. There were five studies including more than 500 participants and the others had a sample size less than 500 participants. Genotype distribution of controls in all studies was consistent with HWE.

**Table 1:** Characteristics of studies included in the meta-analysis.

First Author	Year	Country	Ethnicity	Control	No. of Cases	No. of Controls	Cancer Type	Sample size	Cases			Controls		
									AA	CA	CC	AA	CA	CC
Liu JM	2013	China	Asian	Population Based	375	400	Prostate Cancer	Large	103	172	100	110	196	94
Babar M	2012	UK	Caucasian	Population Based	1070	194	Esophageal Cancer	Large	178	508	384	36	75	83
Du B	2012	China	Asian	Hospital Based	150	180	Nasopharyngeal Carcinoma	Small	34	80	36	40	93	47
Guo JY	2012	China	Asian	Hospital Based	170	160	Colorectal Cancer	Small	49	85	36	42	76	42
Taheri M	2012	Iran	Asian	Population Based	72	93	Breast Cancer	Small	11	32	29	8	45	40
Saenz-Lopez P	2010	Spain	Caucasian	Population Based	154	500	Other Types	Large	19	76	59	73	261	166
Asefi V	2009	Iran	Asian	Hospital Based	111	212	Other Types	Small	15	53	43	29	101	82
Farjadfar A	2009	Iran	Asian	Hospital Based	73	97	Other Types	Small	13	45	15	11	46	40
Haghshenas MR	2009	Iran	Asian	Population Based	142	311	Colorectal Cancer	Small	15	72	55	48	144	119
Haghshenas MR	2009	Iran	Asian	Population Based	87	311	Other Types	Small	16	40	31	48	144	119
Khalili-Azad T	2009	Iran	Asian	Population Based	200	206	Breast Cancer	Small	33	103	64	33	97	76
Nong LG	2009	China	Asian	Population Based	250	270	Nasopharyngeal Carcinoma	Large	71	132	47	68	133	69
Samsami DA	2009	Iran	Asian	Hospital Based	85	158	Other Types	Small	12	51	22	26	75	57
Farhat K	2008	Tunisia	African	Population Based	163	164	Nasopharyngeal Carcinoma	Small	28	94	41	34	77	53
Kashef MA	2008	Iran	Asian	Population Based	19	103	Other Types	Small	3	10	6	16	54	33
Qi T	2008	China	Asian	Hospital Based	50	50	Cervical Cancer	Small	28	17	5	9	24	17
Liu Y	2007	China	Asian	Hospital Based	265	280	Prostate Cancer	Large	72	143	50	78	137	65
Nikiteas N	2007	Greece	Caucasian	Population Based	84	89	Colorectal Cancer	Small	18	47	19	22	32	35
Vairaktaris E	2007	Germany	Caucasian	Population Based	149	89	Other Types	Small	28	66	55	22	32	35
Wei YS	2007	China	Asian	Hospital Based	235	250	Esophageal Cancer	Small	64	123	48	67	124	59
Yang HL	2007	China	Asian	Population Based	107	80	Cervical Cancer	Small	24	50	33	36	26	18
Pratesi C	2006	Italy	Caucasian	Population Based	89	130	Nasopharyngeal Carcinoma	Small	21	42	26	23	64	43

### Main results

**Figure 2:**
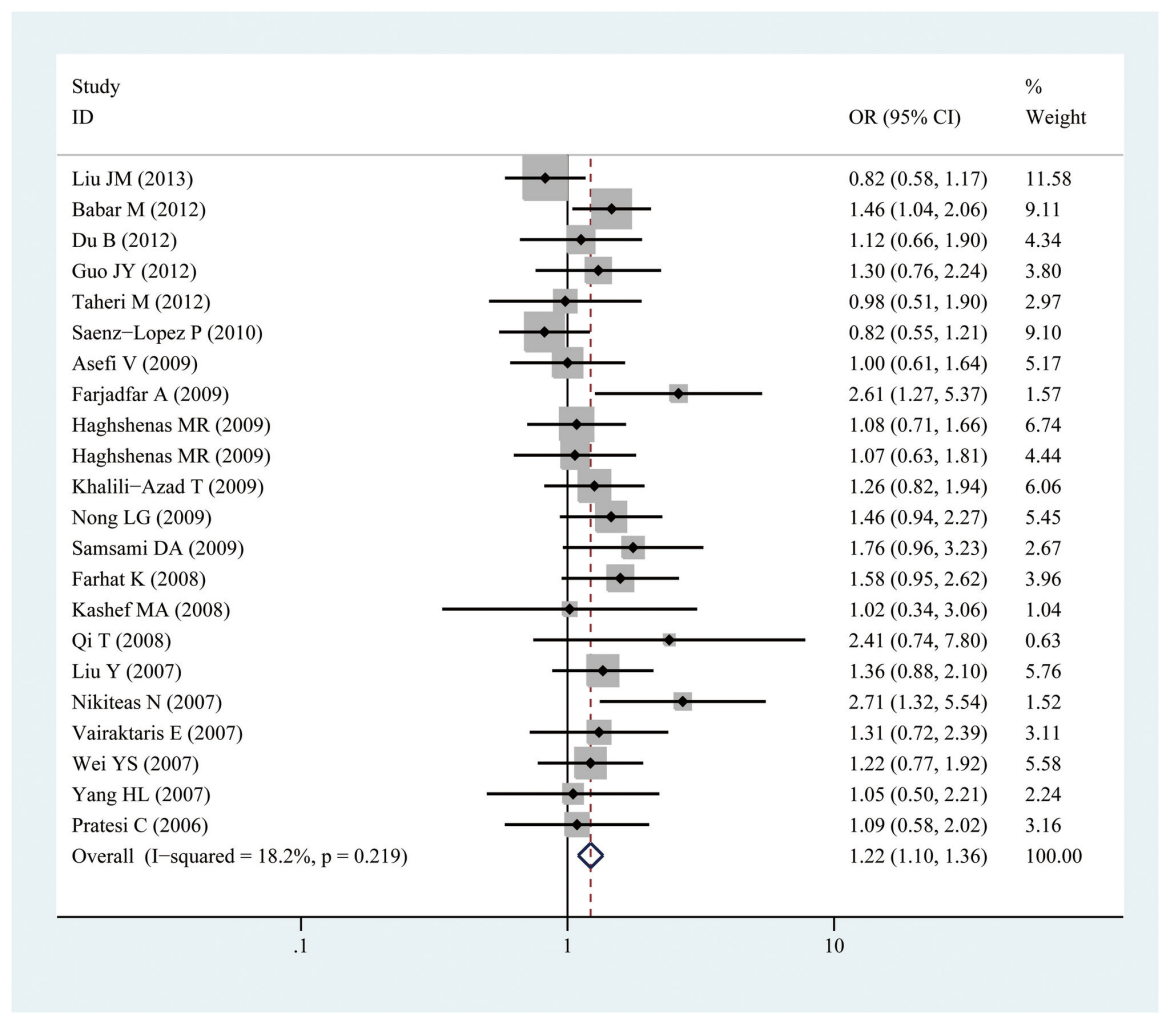
Forest plot of heterozygote comparison for overall comparison (CA vs. CC).

**Figure 3:**
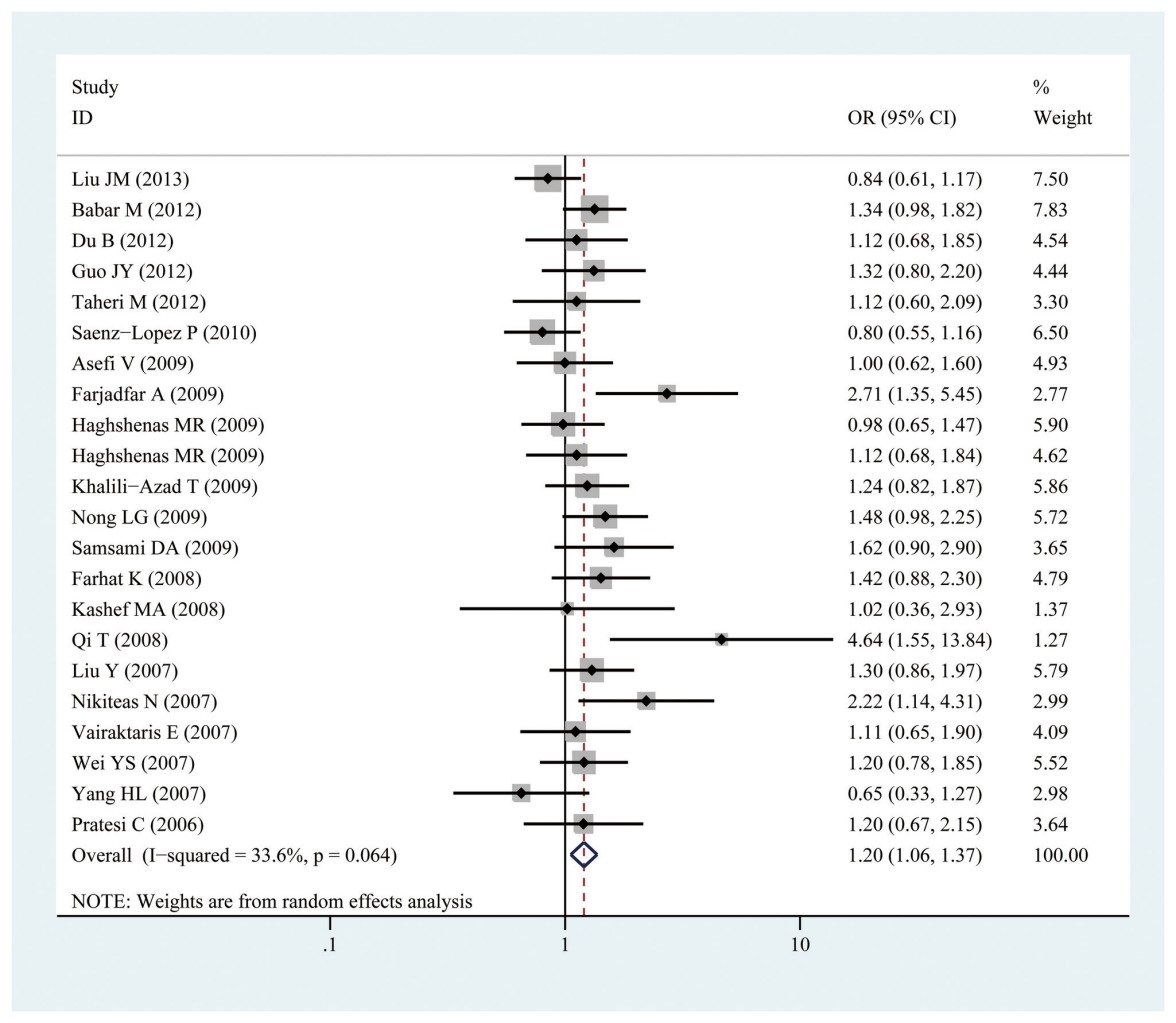
Forest plot of dominant model for overall comparison (CA/AA vs. CC).

Given that the P value of Q-tests was less than 0.10 under the allelic, homozygous, recessive, and dominant genetic models, the random-effects model was used. By contrast, the P value of Q-tests was more than 0.10 under the heterozygous genetic model (P for heterogeneity = 0.219); thus, the fixed-effects model was adopted. Significant associations between -607C/A polymorphisms in IL-18 gene promoter and cancer risk were observed in the heterozygous model (CA vs CC:OR =1.221, 95% CI: 1.096, 1.360; P_heterogeneity_=0.219, Figure 2) and the dominant model (AA/CA vs. CC:OR =1.203, 95% CI: 1.057, 1.369; P_heterogeneity_=0.064, Figure 3) in this meta-analysis. However, no significant association between -607C/A polymorphisms in IL-18 gene promoter and cancer risk was observed under the allelic model(A vs C:OR =1.088, 95% CI: 0.987,1.200; P_heterogeneity_=0.003), homozygous model(AA vs CC:OR =1.139, 95% CI: 0.948, 1.369; P_heterogeneity_=0.023), and recessive model (AA vs. CC/CA: OR =0.995, 95% CI: 0.851, 1.163; P_heterogeneity_=0.025) (shown in Table 2).

**Table 2:** Stratified analyses of the -607C/A polymorphisms in IL-18 gene promoter with cancer risk.

	A vs. C			AA vs. CC			CA vs. CC			AA vs. CC/CA			AA/CA vs. CC		
	Study	OR(95% CI)	Phet	Study	OR(95% CI)	Phet	Study	OR(95% CI)	Phet	Study	OR(95% CI)	Phet	Study	OR(95% CI)	Phet
Overall	23	1.088 (0.987-1.200)	0.003	23	1.139 (0.948-1.369)	0.023	23	1.221 (1.096-1.360)*	0.219	23	0.995 (0.851-1.163)	0.025	23	1.203 (1.057-1.369)*	0.064
Ethnicity															
Asian	17	1.107 (0.972-1.260)	0.001	17	1.196 (0.936-1.530)	0.007	17	1.191 (1.047-1.356)*	0.487	17	1.035 (0.854-1.255)	0.011	17	1.197(1.023,1.401)*	0.088
Caucasian	5	1.041 (0.909-1.193)	0.336	5	1.023 (0.779-1.343)	0.473	5	1.294 (0.906-1.848)	0.041	5	0.890 (0.696-1.138)	0.620	5	1.198 (0.888-1.618)	0.083
African	1	1.076 (0.790-1.464)	NA	1	1.065 (0.558-2.030)	NA	1	1.578 (0.951-2.620)	NA	1	0.793 (0.455-1.382)	NA	1	1.421 (0.877-2.301)	NA
Source of controls															
Hospital based	9	1.247 (1.022-1.523)*	0.005	9	1.329 (0.924-1.912)	0.009	9	1.353 (1.115-1.642)*	0.435	9	1.135 (0.871-1.479)	0.044	9	1.362(1.134,1.635)*	0.116
Population based	14	1.021 (0.941-1.107)	0.124	14	1.012 (0.832-1.231)	0.196	14	1.165 (1.024-1.327)*	0.189	14	0.911 (0.764-1.087)	0.154	14	1.114(0.986,1.258)	0.190
Sample size															
Small	18	1.092 (0.955-1.249)	<0.001	18	1.149 (0.896-1.472)	0.008	18	1.223 (1.036-1.445)*	0.085	18	1.006 (0.813-1.246)	0.008	18	1.200 (1.006-1.430)*	0.016
Large	5	1.032 (0.918-1.161)	0.269	5	1.051 (0.836-1.320)	0.313	5	1.134 (0.863-1.490)	0.042	5	0.980 (0.824-1.164)	0.810	5	1.107 (0.862-1.421)	0.052
Cancer types															
Prostate cancer	2	0.993 (0.852-1.157)	0.385	2	0.993 (0.732-1.346)	0.331	2	1.039 (0.639-1.690)	0.081	2	0.985 (0.773-1.254)	0.896	2	1.027 (0.675-1.565)	0.109
Esophageal cancer	2	1.095 (0.926-1.293)	0.852	2	1.111 (0.799-1.544)	0.783	2	1.371 (1.045-1.800)*	0.528	2	0.945 (0.713-1.253)	0.591	2	1.289 (1.002-1.658)*	0.700
Nasopharyngeal carcinoma	4	1.144 (0.985-1.328)	0.845	4	1.305 (0.961-1.772)	0.759	4	1.330 (1.029-1.719)*	0.704	4	1.082 (0.842-1.391)	0.547	4	1.323 (1.037-1.687)*	0.823
Colorectal cancer	3	1.066 (0.883-1.286)	0.262	3	1.092 (0.664-1.795)	0.213	3	1.460 (0.898-2.371)	0.097	3	0.896 (0.638-1.259)	0.359	3	1.337 (0.865-2.068)	0.118
Breast cancer	2	1.147 (0.904-1.456)	0.726	2	1.332 (0.800-2.216)	0.438	2	1.169 (0.814-1.678)	0.532	2	1.225 (0.716-2.096)	0.274	2	1.204 (0.854-1.696)	0.784
Cervical cancer	2	1.396 (0.208-9.382)	<0.001	2	1.890 (0.069-51.901)	<0.001	2	1.397 (0.644-3.031)	0.241	2	1.403 (0.090-21.882)	<0.001	2	1.653 (0.241-11.339)	0.003
Other cancers	8	1.044 (0.910-1.196)	0.200	8	0.978 (0.717-1.334)	0.223	8	1.100 (0.803-1.507)	0.014	8	0.935 (0.738-1.183)	0.804	8	1.075 (0.795-1.454)	0.012

OR: odds ratio; CI: confidence intervals; Phet: P value for heterogeneity; * OR with statistical significance

### Subgroup analyses, sensitivity analysis and cumulative meta-analysis

**Figure 4:**
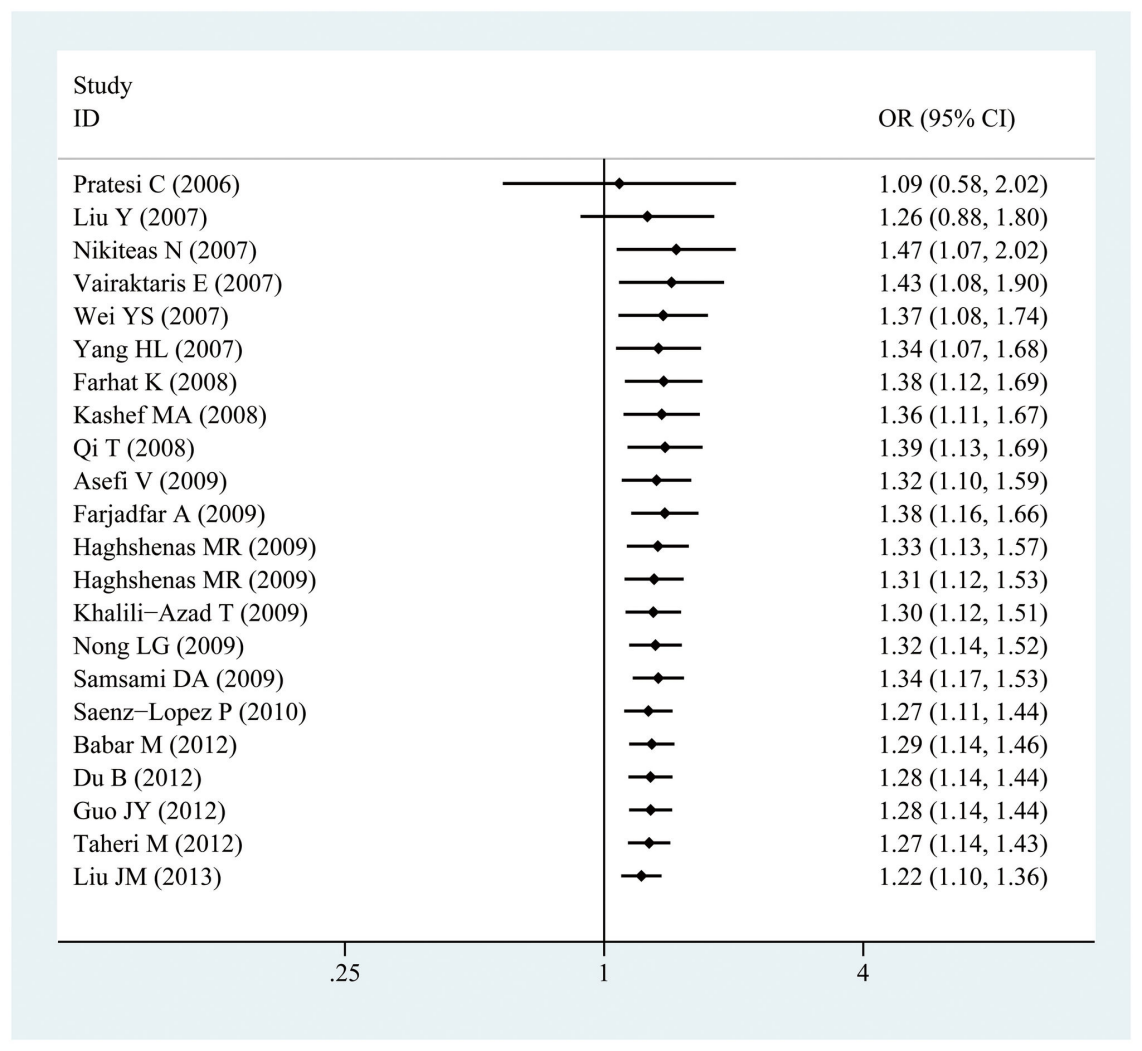
Cumulative meta-analysis of association between -607C/A polymorphisms in IL-18 gene promoter and cancer risk under the heterozygous model(CA vs. CC).

In a stratified analysis by specific cancer types, -607C/A polymorphisms in IL-18 gene promoter was significantly associated with an increased risk of nasopharyngeal carcinoma (CA vs CC:OR =1.330, 95% CI: 1.029,1.719; P_heterogeneity_=0.704; AA/CA vs. CC:OR =1.323, 95% CI: 1.037,1.687; P_heterogeneity_=0.823) and esophageal cancer(CA vs CC:OR =1.371, 95% CI: 1.045,1.800; P_heterogeneity_=0.528; AA/CA vs. CC:OR =1.289, 95% CI: 1.002,1.658; P_heterogeneity_=0.700) in the heterozygous model and dominant model. No evidence of association was found in any genetic model between-607C/A polymorphisms in IL-18 gene promoter and the risk of prostate cancer, colorectal cancer, breast cancer, cervical cancer, and other cancers(shown in Table 2). According to ethnicity, the polymorphism presented a significantly increased risk of cancer among Asian population in the heterozygous model and dominant model(CA vs CC:OR =1.191, 95% CI: 1.047,1.356; P_heterogeneity_=0.487; AA/CA vs. CC:OR =1.197, 95% CI: 1.023,1.401; P_heterogeneity_=0.088); however, no significant association was found in Caucasian and African population (shown in Table 2). In the stratified analysis by source of control groups, we found that the -607C/A polymorphisms in IL-18 gene promoter was associated with a significantly increased risk in hospital-based controls in the allelic model (A vs C:OR =1.247, 95% CI: 1.022, 1.523; P_heterogeneity_=0.005), heterozygous model(A vs C:OR =1.353, 95% CI: 1.115, 1.642; P_heterogeneity_=0.435), and dominant model(A vs C:OR =1.362, 95% CI: 1.134,1.635; P_heterogeneity_=0.116). However, among studies with population-based controls, a significant association was only observed in heterozygous model(A vs C:OR =1.165, 95% CI: 1.024, 1.327; P_heterogeneity_=0.189). When stratifying the sample size, a significant association was observed among studies with small sample size in the heterozygous model and dominant model (CA vs CC:OR =1.223, 95% CI: 1.036,1.445; P_heterogeneity_=0.085; AA/CA vs. CC:OR =1.200, 95% CI: 1.006,1.430; P_heterogeneity_=0.016), but not observed among studies with large sample size in any genetic models. To test the robustness of association, sensitivity analysis was carried out by excluding studies one-by-one and analyzing effect size for all of rest studies. Sensitivity analysis indicated that no significant variation in combined RR by excluding any of the study, confirming the stability of present results. Cumulative meta-analyses were carried out in the heterozygous and dominant genetic models. Between 2006 and 2013, with each accumulation of more studies, the 95% CIs for the pooled ORs became increasingly narrower, indicating that the precision of the estimation was progressively boosted by continually adding more samples(shown in Figure 4).

### Meta-regression and Publication bias

**Figure 5:**
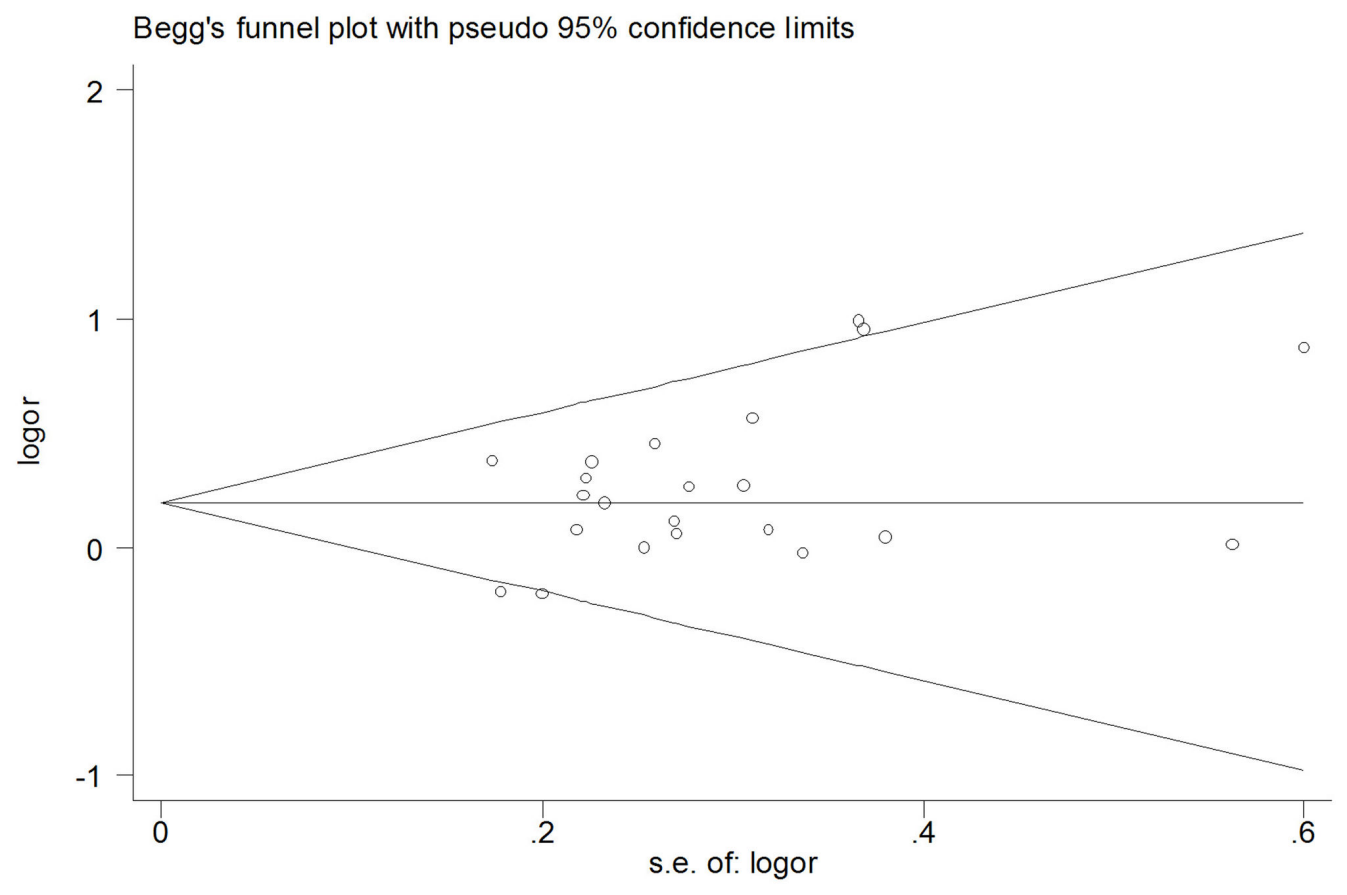
Funnel plot for publication bias in the studies investigating the association between -607C/A polymorphisms in IL-18 gene promoter and risk of cancer(heterozygous genetic model: CA vs. CC). No publication bias was observed among studies using Begg’s P value (P = 0.167) and Egger’s (P = 0.387) test, which suggested there was no evidence of publication bias.

As shown in Table 2, significant heterogeneity was present in all models except for heterozygous model, hence, meta-regression was conducted to detect the source of heterogeneity. Ethnicity, source of controls, sample size and cancer type, which may be potential sources of heterogeneity, were tested by a meta-regression method. The results showed that, in the dominant model (AA/CA vs. CC) for instance, the heterogeneity could only be explained by cancer type(p=0.014), but not ethnicity, sample size, or the source of controls. The potential publication bias of the literatures was evaluated by funnel plot and Egger’s test. No visual publication bias was found in the funnel plot (Figure 5). And Egger’s test suggested that no publication bias was detected in all the comparison models (P >0.05)

## Discussion

The present meta-analysis, which included 4100 cancer cases and 4327 controls from 21 publications with 22 case-control studies, explored the relationship between -607C/A polymorphisms in IL-18 gene promoter and cancer risk. For overall comparison of pooled ORs, significantly increased risk was observed in the heterozygous model(CA vs CC) and the dominant model(AA/CA vs. CC). Under the allelic, homozygous and recessive genetic models, there was no significant association between -607C/A polymorphisms in IL-18 gene promoter and cancer risk. Overall, a significant association exists between -607C/A polymorphisms in IL-18 gene promoter and cancer risk. This finding indicates that the genetic variant in IL-18 gene promoter region may crucially modify the susceptibility of cancers. The C to A substitution at position −607 disrupts a consensus cAMP-responsive element protein-binding site, causing altered transcription factor binding and gene expression [9]. IL-18 serum levels have been reported to be elevated in a variety of cancers compared with control group [19,36-39]. Hence, the -607C/A polymorphisms in IL-18 gene promoter may modify the susceptibility of cancers though changing the expression of IL-18 gene. The mechanism needs further investigation.

When identifying eligible studies by reading full text, the study conducted by Jaiswal PK and colleagues [40] was excluded for incorrect data. The OR and 95% CI under heterozygous genetic model (OR =0.59, 95% CI: 0.39, 0.92)we got based on the genotype frequency in cases and controls(CC: 81, CA: 89 in cases; CC: 61, CA: 113 in controls) were totally opposed to that they got(OR =1.59, 95% CI: 1.01-2.95). Hence, we excluded this study for its unbelievable result.

In the stratified analysis based on ethnicity, a significant increased risk of cancer was found in Asian population, but not in Caucasian or African population. One probable reason is that different environment they live in and different genetic backgrounds may account for these differences. As we know, different populations carry different genotype and/or allele frequencies of this locus polymorphism and may lead to various degrees of cancer susceptibility [41]. And different ethnic groups live with multiple life styles and environmental factors and thus yield diverse gene-environment interactions [42]. In addition, there are only one study and five studies investigating the association between -607C/A polymorphisms in IL-18 gene promoter and cancer risk among African and Caucasian population, respectively. Insufficient number of patients limited us to detect stable effects in these two populations. Additional studies are warranted to further validate ethnic difference in the effect of -607C/A polymorphisms in IL-18 gene promoter on cancer risk, especially in Africans. During sub-group analyses, we found that the source of controls also affected the association between -607C/A polymorphisms in IL-18 gene promoter and cancer risk. A significant association was observed in hospital-based controls under allelic and dominant genetic models, but not the population-based controls. The reason may be that the hospital-based studies have some inherent selection biases as such controls may just represent a sample of ill-defined reference population and may not be very representative of the study population or the general population. In stratified analysis by cancer site, we found that -607C/A polymorphisms in IL-18 gene promoter was statistically related with an increased risk of esophageal cancer and nasopharyngeal carcinoma. However, no evidence of association was found in any genetic model between-607C/A polymorphisms in IL-18 gene promoter and the risk of prostate cancer, colorectal cancer, breast cancer, cervical cancer, or other cancers. One possible reason is that carcinogenic mechanism underlying the etiology may differ by different tumor sites and that the -607C/A polymorphisms in IL-18 gene promoter may play a different role in different cancers. Futher, the number of studies which investigated the association between -607C/A polymorphisms in IL-18 gene promoter and risk of different types of cancer was too small(≤3), which limited us to detect stable effects on different cancer types. So, more studies focusing on different cancer types are need in the future.

The strength of the present analysis lies in inclusion of 22 studies, reporting data of 4100 cancer cases and 4327 controls. Publication bias, which, due to the tendency of not publishing small studies with null results, was not found in our meta-analysis. Furthermore, our findings were stable and robust in sensitivity analyses. Cumulative meta-analyses showed that, with each accumulation of more studies, the 95% CIs for the pooled ORs became increasingly narrower, indicating that the precision of the estimation was progressively boosted by continually adding more samples. Some limitations might be included in the meta-analysis. Firstly, we did not search for unpublished studies, so only published studies were included in our meta-analysis. Therefore, publication bias may have occurred although no publication bias was indicated from both visualization of the funnel plot and Egger’s test. Secondly, the results were based on unadjusted ORs, while a more precise estimation should take into account the effect of multiple confounders such as age, smoking status, drinking status and environmental factors on the association. Lack of information for data analysis may cause serious confounding bias. Thirdly, the small sample size is the major defect in this meta-analysis. In the stratified analysis by ethnicity and cancer type, the sample size of studies among Caucasians, Africans and among several cancer types is small, which limited us to detect stable effects in these populations and cancer types. Further studies are warranted to further evaluate the association in different ethnicities and cancer types in the future. Additionally, heterogeneity was significant in our meta-analysis, which may attenuate the strength of this study.

In conclusion, the present meta-analysis suggests that the -607C/A polymorphisms in IL-18 gene promoter is associated with a significantly increased risk of cancer, especially for nasopharyngeal carcinoma and esophageal cancer and in Asian population. More studies with larger sample size, well controlled confounding factors are warranted to further evaluate the association in different ethnicities and different cancer types in the future.

## Supporting Information

Checklist S1 **PRISMA checklist.**
(DOC)
